# Supplementary material for: Purcell-enhanced single photons at telecom wavelengths from a quantum dot in a photonic crystal cavity
Source: Sci Rep. 2024 Feb 23;14:4450. doi: 10.1038/s41598-024-55024-6 (PMC11310300; doi:10.1038/s41598-024-55024-6)
Supplement: Supplementary file 1 — Supplementary Information. [file 41598_2024_55024_MOESM1_ESM.pdf]

Supplementary Information - Purcell-Enhanced Single Photons at Telecom Wavelengths from a Quantum Dot in a Photonic Crystal Cavity

Catherine L. Phillips[1,*], Alistair J. Brash[1], Max Godsland[2], Nicholas J. Martin[1], Andrew Foster[1], Anna Tomlinson[1], René Dost[1], Nasser Babazadeh[2], Elisa M. Sala[2], Luke Wilson[1], Jon Heffernan[2], Maurice S. Skolnick [1], and A. Mark Fox[1]

[1]Department of Physics and Astronomy, University of Sheffield, UK
[2]EPSRC National Epitaxy Facility, Department of Electronic and Electrical Engineering, University of Sheffield, UK
[*]c.l.phillips@sheffield.ac.uk

ABSTRACT

Supplementary information including further details of the QD radiative lifetimes measured for τ_{bulk} and the temperature-dependent count rates observed during the second-order correlation Hanbury-Brown and Twiss measurements.

1 Measuring radiative lifetime of QDs in the bulk

To find the Purcell enhancement of the L3 PhCC structure we first obtained a reference radiative lifetime (τ_{bulk}) using QDs in a bulk wafer. Non-resonant pulsed excitation at 850 nm was used to measure the reference radiative lifetime (τ_{bulk}) for three QDs in the bulk wafer, the wavelength and measured lifetimes for these QDs is detailed in Supplementary Table 1. A fourth QD in the bulk was measured using quasi-resonant p-shell excitation. Here, the QD was excited at a detuning of 20 meV with a pulse length of 8 ps using a PriTel Ultrafast Optical Clock. The p-shell radiative lifetime lies within the range measured using above band excitation. Using the mean of the values listed in Supplementary Table 1 gives a τ_{bulk} of 1.86 ± 0.22 ns.

Wavelength (nm)	τ_{bulk} (ns)
1488.9	1.72 ± 0.01
1497.7	1.64 ± 0.01
1529.1	2.10 ± 0.05
1592.1 *	1.98 ± 0.02

Supplementary Table 1. Wavelength and radiative lifetime (τ_{bulk}) of QDs in the bulk InP membrane, away from cavity structures. For the first three QDs, τ_{bulk} was measured using above band excitation, the fourth (marked *) was measured using p-shell quasi-resonant excitation.

2 Temperature-dependent count rates

Here we present the temperature-dependent average count-rate per detector, as measured by SNSPDs during the three second-order correlation Hanbury-Brown and Twiss (HBT) measurements of the cavity-coupled QD. We observe no clear trend in count rate as the temperature is increased under constant excitation conditions. This consistency in count rates is a clear indicator that any non-radiative losses are negligible in this temperature range.

Temperature (K)	Counts (kcps)
4	10
20	14
25	12

Supplementary Table 2. Temperature-dependent count rates on the measured on the SNSPDs during the second-order correlation Hanbury-Brown and Twiss measurements shown in Figure 4.
